# Urban seismic resilience mapping: a transportation network in Istanbul, Turkey

**DOI:** 10.1038/s41598-022-11991-2

**Published:** 2022-05-17

**Authors:** Ji-Eun Byun, Dina D’Ayala

**Affiliations:** 1 Engineering Risk Analysis Group, Technical University of Munich, Munich, Germany; 2 Department of Civil, Environmental and Geomatic Engineering, University College London, London, UK

**Keywords:** Civil engineering, Statistics

## Abstract

When a seismic event occurs, transportation networks play a critical role in undertaking emergency activities such as evacuation and relief supply. Accordingly, to secure their functionality, it is essential to accurately assess their resilience. In particular, this study performs a rigorous probabilistic analysis on the seismic resilience of a transportation network in Istanbul, Turkey. The analysis accuracy is enhanced by considering, along with the structural damage of roadways, the additional disruption mode of network performance caused by the debris falling from damaged objects in their vicinity. Moreover, we obtain the results as a map of resilience measure, which enables us to investigate the disruption inequality across the study area and identify critical factors that govern the system resilience. To enable such sophisticated probabilistic analysis, a Bayesian network (BN) model is developed that involves various types of information from the hazard process to the performance of structures and systems. Then, the BN is quantified by identifying and compiling a comprehensive list of datasets. Thereby, this study analyses large-scale systems involving thousands of structures, while providing general probabilistic models and data schema that can be employed for other transportation networks.

## Introduction

Transportation networks play a critical role in maintaining welfare and economic systems of modern societies^1^. Their role becomes even more critical when facing disruptive events such as earthquakes, as they are essential to enable emergency activities, evacuation and relief supply and to expedite restorations to normality. The breakdown of these systems under a seismic event can bring about devastating consequences, as witnessed, for instance, in the 2016 Kaikoura, New Zealand earthquake^2^. To prevent such disastrous outcomes, it is essential to accurately evaluate the disaster resilience of those systems.

However, such analysis remains challenging as it involves diverse sets of variables interacting to each other (e.g. hazard processes, structural damages and recovery works), a particularly critical issue for large-scale transportation networks with many roadways, such as metropolitan neighbourhoods. Since a resilience analysis requires a modelling and evaluation of the complex dependency between those variables, challenges arise from both compiling all relevant data and computing complex models. Moreover, while the primary disruption would be caused by the structural damage of roadways, further disruptions can occur as the debris generated from the adjacent objects (e.g. buildings, overpasses and slopes) falls onto the roadways^3–5^. Such additional disruption mode heightens the aforementioned challenges, i.e. data compilation and correlation/computational scale. These disruptions may have a significant influence on analysis results, especially for urban transportation networks which are often set within dense built up areas. While this issue has been investigated at the level of individual roadways^2,6^, this is an area of emerging study currently hindered by limited research and data. Still, considering the continuing expansion of urban areas, the analysis model needs to take into account both disruption modes, so that the analysis accuracy can be enhanced, and the interactions across multiple contributing factors can be better understood.

To evaluate system resilience, an essential prerequisite is to define a measure of system functionality. While it can be defined either at component-level or at system-level, the latter definition enables us to consider the interdependency between the operation of roadways and thereby, to reflect the reality more accurately. However, such system-level approach is more challenging as the associated network analysis in general demands more detailed data and higher computational cost. Moreover, since the objective of resilience analysis is to enable risk-informed decision-making, it is critical to establish resilience metrics that effectively serve decision objectives of interest and communicate loss and recovery in operational terms. This study aims to address the inequality in the accessibility of infrastructure, following a seismic event. While it has been actively investigated for transportation planning^7,8^, the relevance of inequality in access and mobility has been recognised only recently in the context of disaster resilience^9,10^.

Real world urban transportation networks are often characterised by a clear hierarchy among roadways depending on their connectivity, traffic volumes, vehicle speeds and accessibility, conveniently categorised into arterials, collectors and local roads^11^. Arterial roads aim to provide high levels of mobility and high speed, and they can be accessed from other roads only through designated entrances. The collectors connect local roads to arterials, by collecting traffic from local roads and distributing it to arterials, or vice versa. Local roads are the roads that define the neighbourhoods, serving residential, commercial and social assets, by carrying lower volumes of traffic at the lowest speed limit. Such limited interactions and different roles across the system make the robustness of connections from local roads to arterial roads crucial, so that, during a hazardous event, residential neighbourhoods can remain accessible to emergency activities and evacuation, relief supplies, health care, etc. As transportation networks underpin the delivery of multiple activities both in the immediate emergency and in the recovery phase, a proper choice should be made on tools and scales of network analysis. Such choice is especially critical for road networks having grown organically over time, where, in contrast to a planned network with a grid topology, connectivity, capacity and vulnerability show high spatial fluctuations at local scale, which affect the system performance and resilience^12^.

In this study, the aforementioned issues are addressed by analysing the seismic resilience of a transportation network in Istanbul, Turkey. The major outcomes of this study are fourfold: First, *a detailed probabilistic model* is set up and evaluated by employing an advanced modelling method, namely Bayesian network (BN)^13,14^. The established model can handle not only complicated dependency between multiple types of variables, but also large-scale transportation networks that can include thousands of structures and roadways. Second, this study identifies, compiles and correlates a comprehensive list of datasets relevant to disaster resilience analysis from both private and open-source databases. The developed *data schema* not only enables detailed analyses but also provides a general guideline on data compilation. Third, the proposed model takes into account both *direct disruptions* (i.e. disruptions caused by the structural damage of roadways) and *indirect disruptions* (i.e. disruptions caused by debris falling from damaged objects in the vicinity). Fourth, analysis results are obtained as *a map of resilience loss* which enables us to spatially visualise and dynamically investigate the disaster inequality across the study area.

In this paper, the terms *structure* and *object* refer to a body that may experience physical damage induced by hazard loads. *Components* indicate the abstract elements that jointly constitute a *system* and make it operational. Because of the specific focus of the current study, the terms *components* and *systems* are used interchangeably with *roadways* and *transportation networks,* respectively. The term *functionality* denotes the extent to which the continued use of those components and the system is preserved, which should be distinguished from the physical damage of structures. Then, *resilience* evaluates the varying functionality over time. As this study deals with seismic risk, *hazards* are confined to *earthquake;* and this also applies to the term *disaster* which refers to a hazard event that actually leads to catastrophic consequences.

## Case study network

### Site

The case study site is located in Istanbul, Turkey, known for high seismic risks as it lies in one of the most seismically active regions of the world^15,16^. Since the two consecutive events, the Izmit (Kocaeli) earthquake of 17 August 1999 and the Duzce earthquake of 12 November 1999^17^ that resulted in thousands of casualty and large population displacement in the Marmara Sea region, the seismic risk in Istanbul has been actively studied^18,19^ including momentous projects to implement mitigation actions^20^. As a continuation of such efforts, this study evaluates the seismic resilience of the transportation network in the city. The current study is developed within the framework of the UKRI GCRF Urban Disaster Risk Hub, Tomorrow’s Cities (https://comet.nerc.ac.uk/tomorrows-cities-istanbul/).

**Figure 1:**
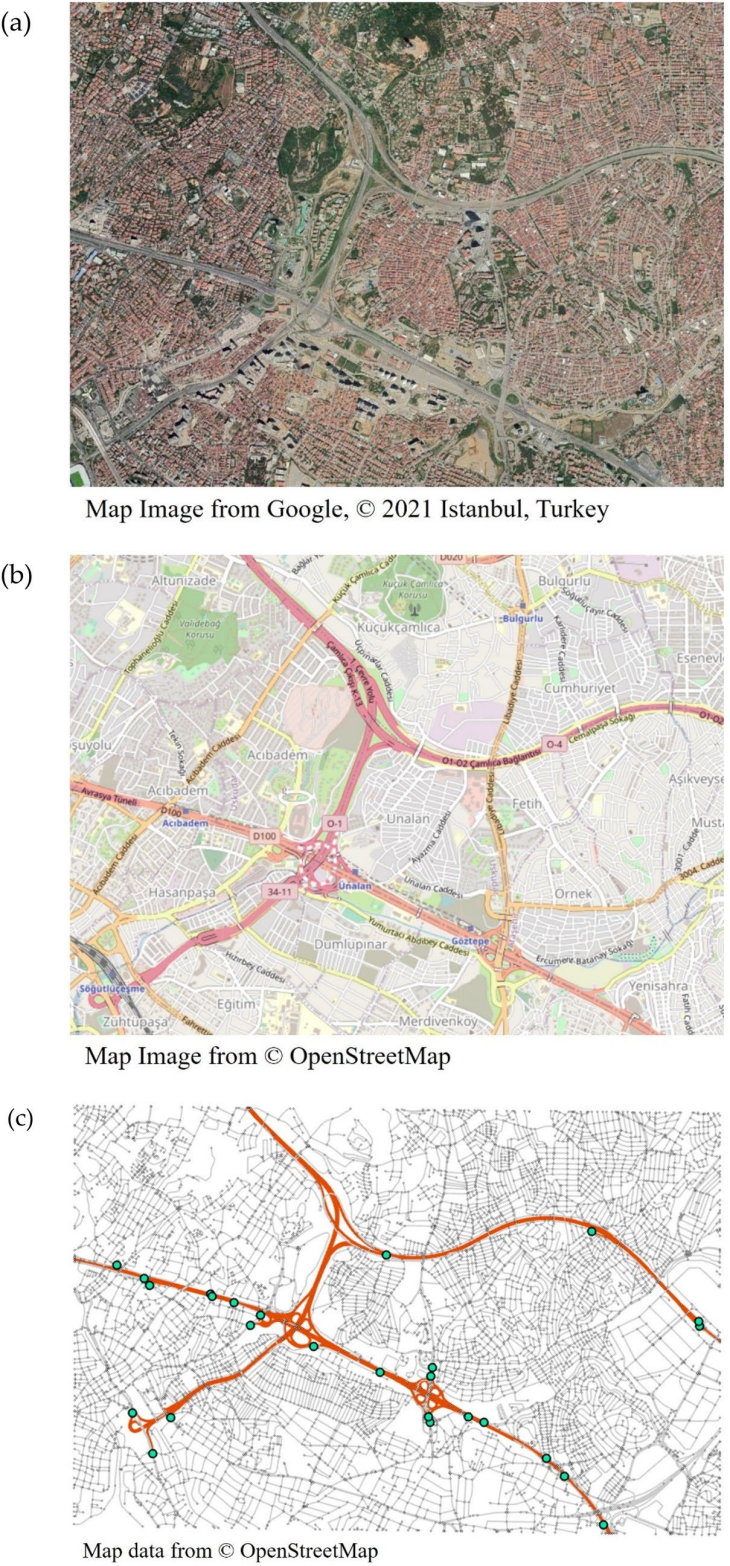
Case study network: (**a**) Satellite imagery (Map image from Google © 2021), (**b**) transportation map (Map image from © OpenStreetMap) and (**c**) roads data (Map data from © OpenStreetMap) with arterial roads (thick orange lines), non-arterial roads (thin grey lines) and entrance vertices to arterials (green circles).

While Istanbul is a large metropolis consisting of multiple districts, this study analyses the area around the Fikirtepe neighbourhood, which lies in the districts of Kadıköy and Üsküdar, Istanbul, Turkey. The satellite imagery of the area and roads map data are respectively presented in Fig. 1a and b, covering the area contained between [40.9798–41.0200]° N and [29.0294–29.1084]° E, with dimensions of 6.9 km $\times$ 4.6 km. On one hand, the case study area is characterised by low-income population, housed in high proportion in “informal” buildings (i.e. buildings whose design and construction do not abide to legal design code), which heightens the community’s vulnerability^21^. In particular, those vulnerable buildings are more likely to collapse and therefore, to block the adjacent roadways by generating debris. Such risk of secondary disruption is particularly relevant to the case study area, which is characterised by dense construction of buildings and narrow roads, as observed in the satellite imagery. On the other hand, it is noted that in Istanbul, the major highways such as O-1 and D100 play a crucial role in connecting residential districts to critical facilities and workplaces across the city, transferring the majority of the daily traffic through the metropolitan area^22^. Accordingly, in this case study, the system functionality is defined as the maximum traffic flow accessible from local neighbourhoods to arterial entrances. In other words, while system functionality is evaluated by maximum flow analysis, the functionality would vary from zero (i.e. a given vertex pair is completely disconnected) to the original flow (i.e. the given pair does not experience any traffic disruptions).

Recently, moreover, owing to its transportation accessibility-related advantages (i.e. being a central location, close to public transport interchange nodes and easily accessible to ring roads), the site is facing increasing pressure from developers, in response to which the government launched a rapid urban transformation programme aimed at replacing those low/mid-rise buildings with high-rise buildings on small footprints. Such transformation has substantially increased population density and traffic demand and disrupted the pre-existing streets grid. These vulnerable conditions and the recent transformation underline the dynamics of the urban development within Istanbul, and the importance of understanding the interaction between constructions and natural hazards, so that urban development can be better informed to promote community’s resilience against quantifiable risks. In addition, such rapid transformation might heighten the social inequality in the community as the rising house prices and population density are likely to marginalise the minority into poor living conditions. The resilience map obtained from the proposed BN enables us to investigate these issues so that proper decisions can be made to secure resilience equality among neighbourhoods.

### Metrics of system functionality and resilience

In this study, the system functionality of interest is defined as the accessibility to arterial roads from local sites (the terms and notations used in this section accord with the probabilistic model presented in greater detail in the “Methods” section). To this end, we perform maximum flow analysis from sets of local places, namely *origin vertices*, to access points to arterial roads, namely *destination vertices;* for analysis, local places are selected to cover the study area uniformly. In Fig. 1c, arterial roads and their access points (i.e. destination vertices) are illustrated respectively with thick orange lines and green circles, while non-arterial roads (i.e. urban roads and collectors) are represented by thin grey lines. Then, for each origin vertex $i\in {\mathcal{N}}_{O}$ where ${\mathcal{N}}_{O}$ is the set of origin vertices, the system functionality at time $t,$ $S{F}_{t,i},$ $t=1,\ldots ,T,$ is obtained as the sum of the maximum flow to all destination vertices, i.e.

1 $$SF_{{t,i}} = \sum\limits_{{j \in {\mathcal{N}}_{D} }} {f_{{ij}}^{t} ,\;i \in {\mathcal{N}}_{O} ,}$$

where $T$ is the number of time points being analysed; ${\mathcal{N}}_{D}$ is the set of destination vertices, respectively; and ${f}_{ij}^{t}$ is the maximum flow (veh/h) from vertex $i$ to $j,$ given the traffic capacity of roads at time $t.$

**Figure 2:**
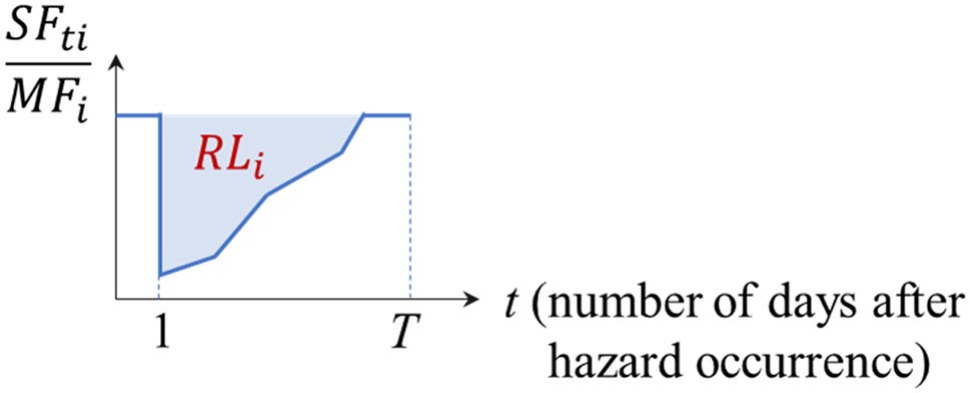
Illustrative graph of the definition of system resilience loss at origin node *i*, ${RL}_{i}$.

Then, the system resilience loss at origin vertex *i*, ${RL}_{i}$ is defined as the total loss of the flows reachable to destination vertices over the analysis time (i.e. the period needed to restore 100% flow), whose figurative definition is illustrated in Fig. 2. To this end, the maximum flow $M{F}_{i}$ is evaluated as

2 $$MF_{i} = \sum\limits_{{j \in {\mathcal{N}}_{D} }} {\bar{f}_{{ij}} ,\;i \in {\mathcal{N}}_{O},}$$

where ${\overline{f} }_{ij}$ refers to the maximum flow from vertex *i* to *j* given the edges with their unmarred capacity. Then, the system resilience loss of each origin vertex $i\in {\mathcal{N}}_{O},$ ${RL}_{i}$

3 $$RL_{i} = \sum\limits_{{t = 1}}^{T} {\left( {1 - \frac{{SF_{{t,i}} }}{{MF_{i} }}} \right),i \in {\mathcal{N}}_{O}}.$$

In other words, as the value of ${RL}_{i}$ increases, the corresponding location would have, during a seismic event, less accessibility to arterial roads.

In detail, the proposed resilience loss measure estimates the system’s functionality loss as a ratio so that the measure can reflect the level of degradation compared to an undisrupted condition. On the other hand, the measure evaluates the cumulative ratio from hazard occurrence to full recovery, in order to capture the total loss of traffic flow. As a result, the resilience loss has the unit of time, which can be regarded as the lost time of the system’s functioning, e.g. 50% loss of 2 days and 25% loss of another 2 days lead to 1.5 days loss in total. This resilience measure allows to quantify the loss in a uniform way across the road network irrespective of the flow capacity of a particular component of length of a particular path between any OD pair.

## Results

The transportation network of the case study area is modelled as a graph using the roads data obtained from the Istanbul Metropolitan Municipality (IMM), which is as illustrated in Fig. 1c. Since the analysis considers the accessibility to arterial roads from local sites, only non-arterial roads are considered during analysis, leading to 10,117 roads and 7133 intersections, i.e. the graph for analysis consists of 10,117 edges and 7133 vertices. The 26 destination vertices, i.e. the entrances to arterial roads, are identified using the road map provided by OpenStreetMap, as marked by green circles in Fig. 1c. On the other hand, the analysis considers all of the 36,921 buildings within the site, individually (for more details, readers are referred to “Methods” section). To visualise the analysis results that are obtained for discrete origin vertices, the computed resilience loss values are interpolated using the inverse distance weighting (IDW) tool available by ArcGIS Pro software.

**Figure 3:**
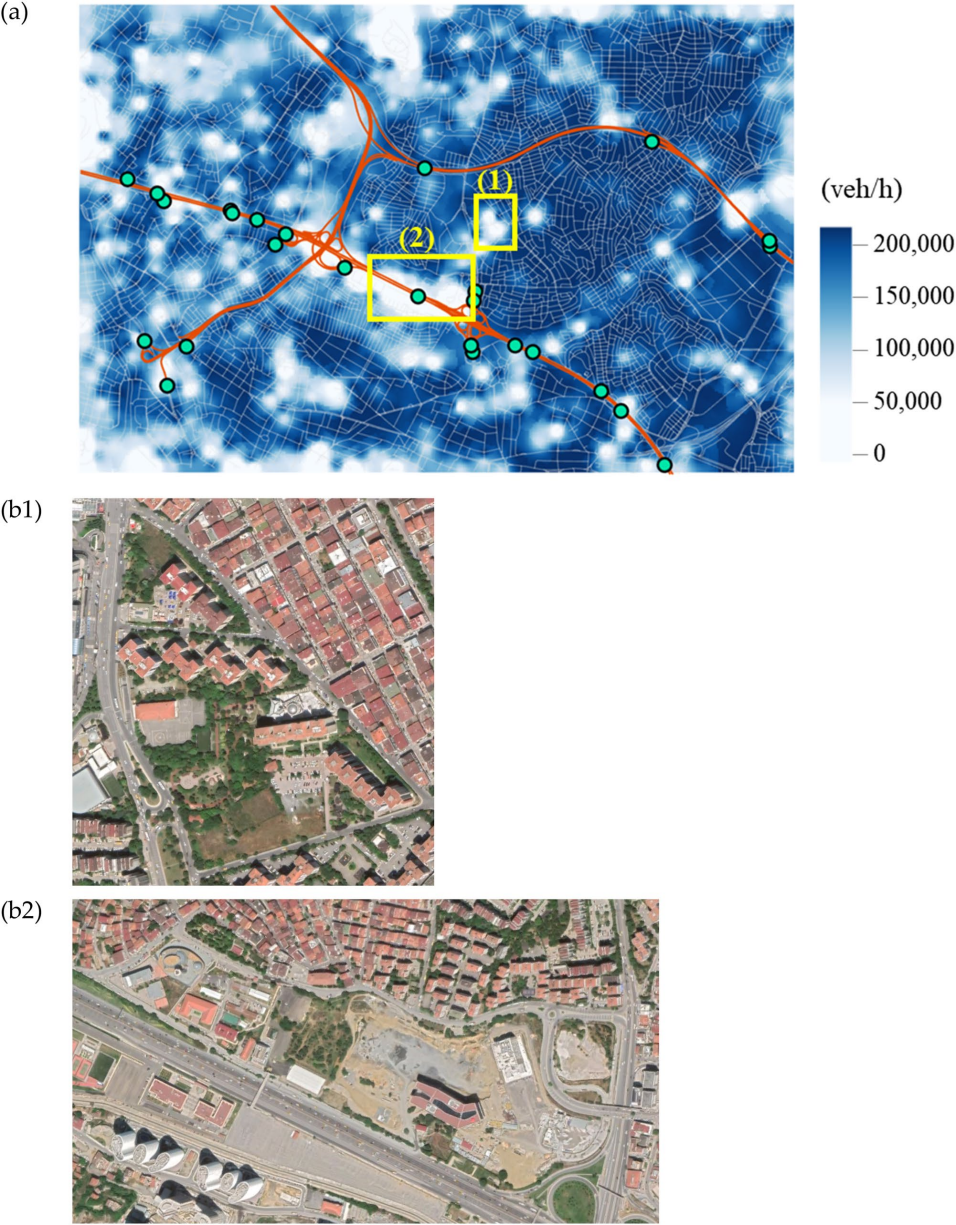
Maximum flows to arterial entrances given undamaged roadways: (**a**) sum of $M{F}_{i}$ in Eq. (2) and (**b**) example sites with particularly bright colours in (**a**), which are marked by yellow boxes in the figure (imagery provided by Google Earth): (**b1**) Green areas and (**b2**) university compound.

To evaluate the resilience loss measure, the maximum flows to arterial entrances, $M{F}_{i}$ in Eq. (2), are required, which is evaluated as in Fig. 3a. It is found that these values mostly depend on the number of entrances that can be reached from each local site and the number of paths available to those entrances. In the map, the places with exceptionally low accessibility (i.e. those with almost white colours) are the locations of green places or complexes such as schools, hospitals and stadiums, which have only a few paths (mostly a single one) connected to outside. For illustration, the satellite imagery of the example locations that are marked with yellow boxes in the figure, are presented in Fig. 3b1,b2. It is noted that since the resilience loss measure, defined in Fig. 2, evaluates only the functionality degradation compared to the original performance, locations with brighter colours in Fig. 3a (i.e. a lower accessibility given undamaged structures) are not necessarily subjected to a higher resilience loss.

**Figure 4:**
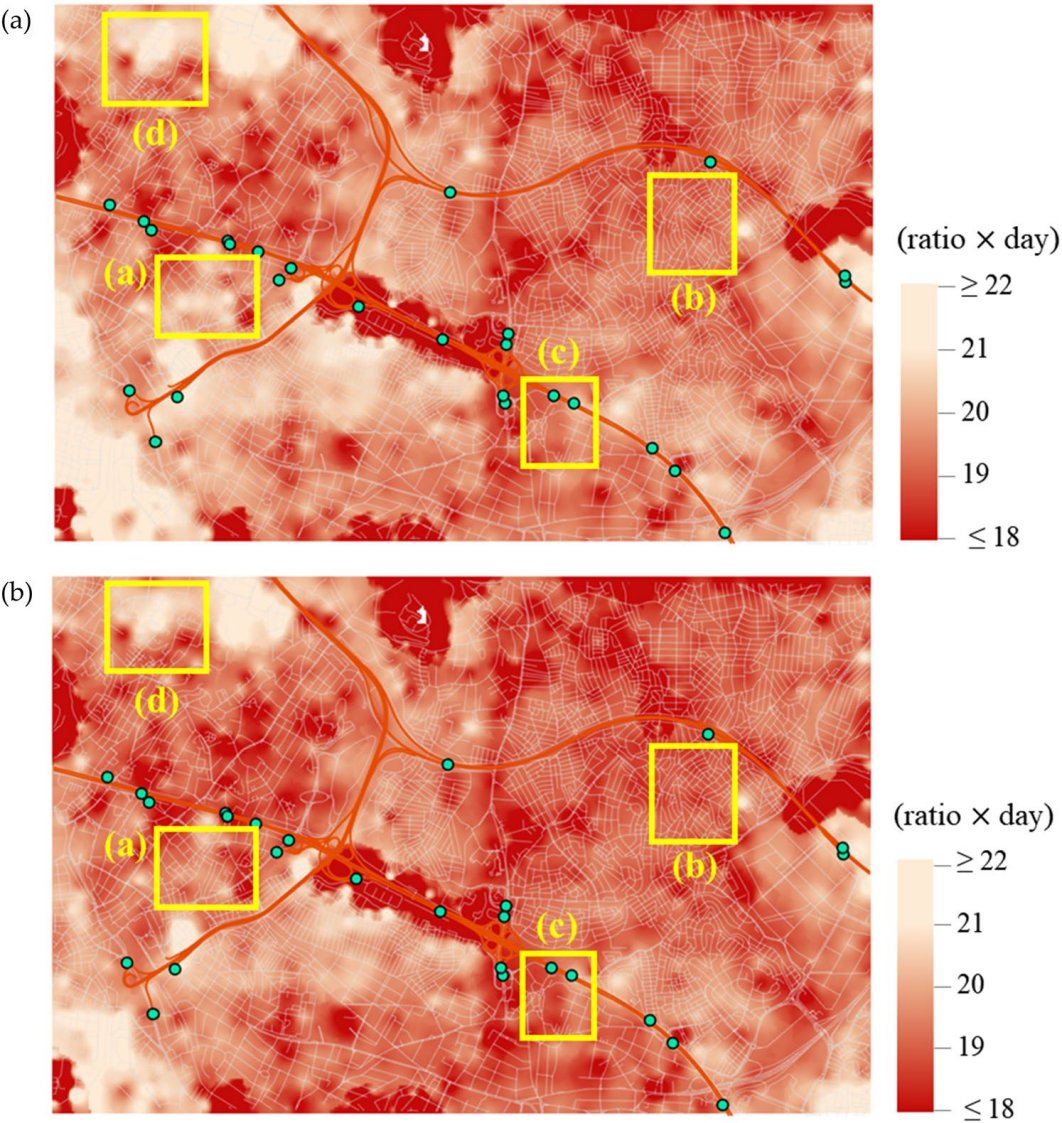
Spatial distribution of system-level resilience loss by sample mean $\widehat{E}\left[{RL}_{i}\right],$ with edges subjected to (**a**) direct and indirect disruptions and (**b**) only direct disruptions.

**Figure 5:**
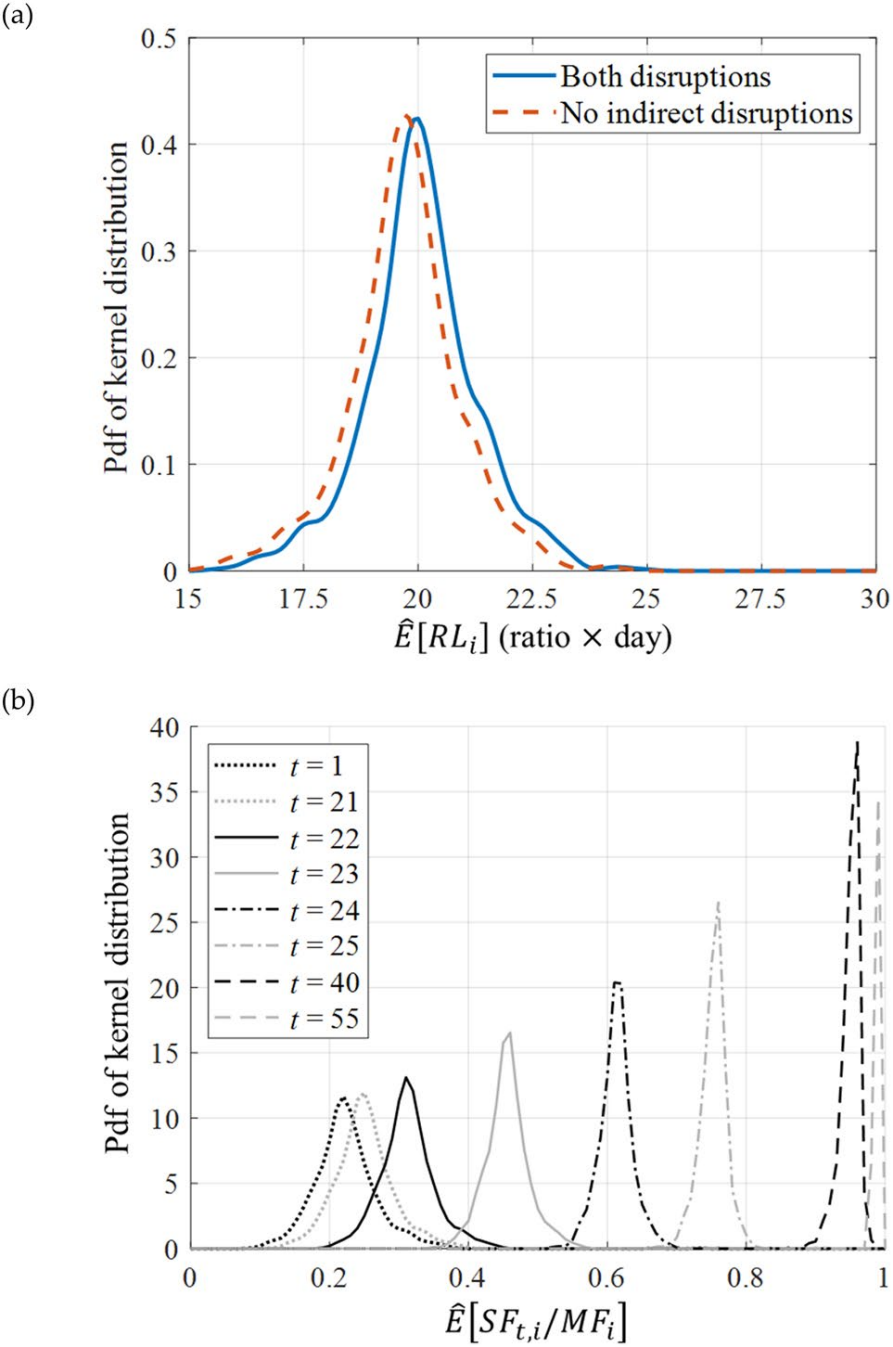
Analysis results fitting: (**a**) Kernel distribution of sample mean $\widehat{E}\left[{RL}_{i}\right]$ for $i\in {\mathcal{N}}_{O},$ with edges subjected to both direct and indirect disruptions (blue solid line) and only direct disruption (orange dashed line) and (**b**) kernel distribution of sample mean $\widehat{E}\left[S{F}_{t,i}/M{F}_{i}\right]$ for $i\in {\mathcal{N}}_{O},$ given *t* = 1, 21 to 25, 40 and 55 for direct and indirect disruptions.

The system-level resilience loss at origin vertex $i\in {\mathcal{N}}_{O}$, ${RL}_{i}$ in Eq. (3) is evaluated as in Fig. 4a. This shows the interpolated map using the MCS sample mean for each vertex $i,$ i.e. $\widehat{E}\left[{RL}_{i}\right].$ From the definition given in Eq. (3), the map has the unit equivalent to the number of days of functionality loss, i.e. (ratio) $\times$ (day), and is visualised within the range of 18 to 22 days, which includes the sample means of most areas. In Fig. 5a, the distribution of the sample mean values for different $i\in {\mathcal{N}}_{O}$ is fitted to a kernel distribution. In the figure, the varying sample means across different locations indicate the inequal resilience over the study area, while the disparity is expected to be widened as the analysis area becomes larger. For example, among central locations, neighbourhoods around area (a) in Fig. 4a show a high resilience loss. This is in agreement with the topological and socio-economic situation of these neighbourhoods, as their urban fabric was developed without predefined planning, and they are occupied by low-income populations housed in poorly constructed buildings with high density, and poor provision of essential infrastructures such as roadways. The recent redevelopment, which sees the construction of high rises along the major arteries, creates a further barrier to connectivity by disrupting the previous road grid, as can be seen in Fig. 4a in the area immediately south of the highway D100, between boxes (a) and (c). The effect of building collapse (indirect disruption) in this area is particularly high as it can be seen by comparing Fig. 4a and b.

To understand the recovery process of the system, the ratio $S{F}_{t,i}/M{F}_{i}$ (i.e. the *y*-axis of Fig. 2 that defines resilience loss) is investigated in Fig. 5b, where the sample means $\widehat{E}\left[S{F}_{t,i}/M{F}_{i}\right],$ $i\in {\mathcal{N}}_{O},$ are fitted to a kernel distributions for *t* = 1, 21, 22, 23, 24, 25, 40 and 55 days. The result shows that the variance in the distribution becomes narrower as $t$ becomes larger, while for more than 95% of the origin vertices, the average number of days required for full recovery is no greater than 55 days (i.e. $t\le 55)$. It is also found that the recovery process is divided into largely two phases around $t=20$ days. Such boundary arises from the average number of days required to repair extensively damaged roadways, which is 21 days (as summarised in Appendix D of Supplementary Information), underlining the importance of this parameter in terms of system recovery. The period of 1–20 days is controlled by debris clearance works and repair works of roadways with minor damage. On the other hand, the long-term recovery process with $t\ge 40$ is governed by the repair of severely damaged bridges.

**Figure 6:**
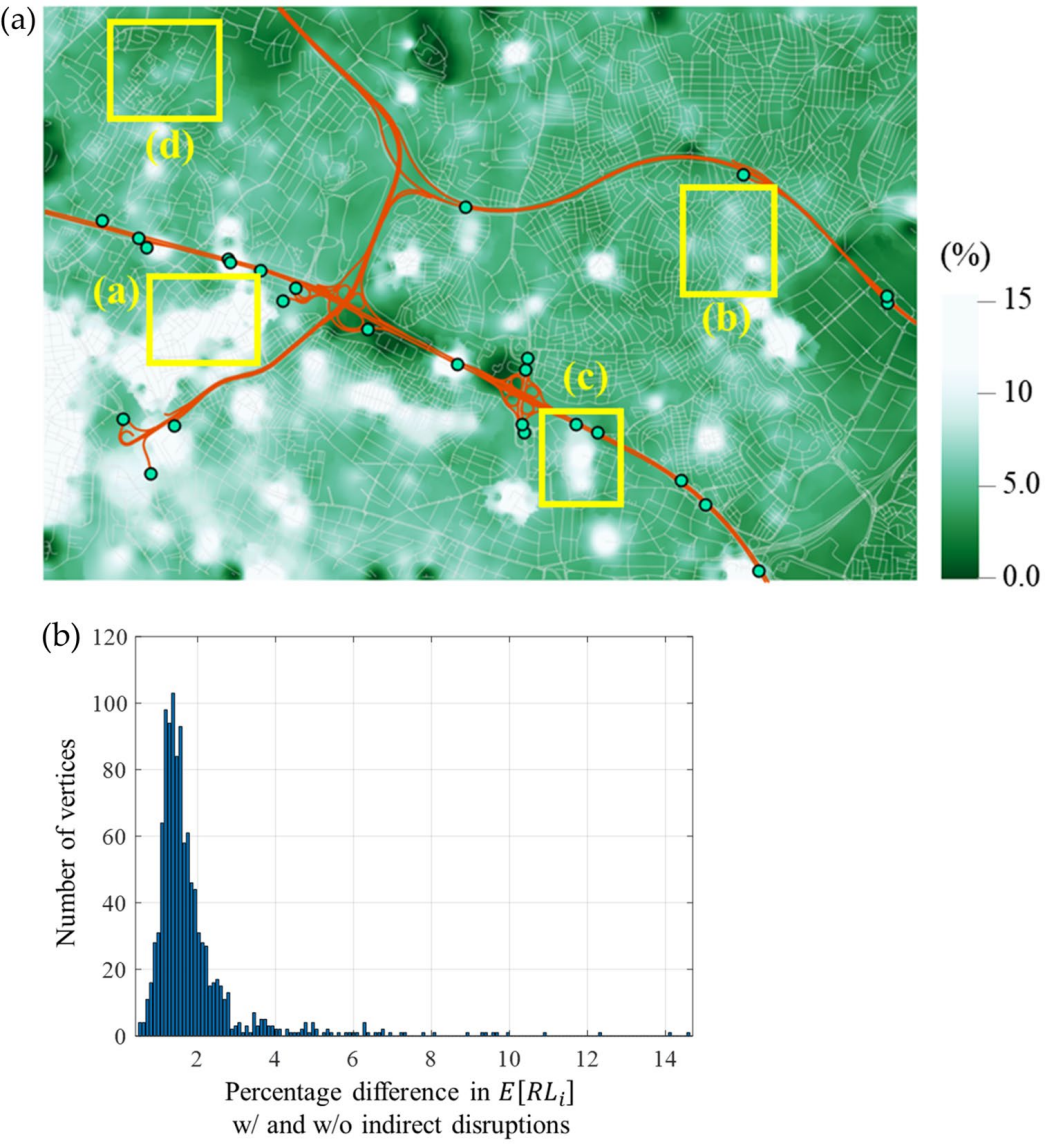
Percentage difference in the mean resilience loss measures computed with and without indirect disruptions: (**a**) Map visualisation and (**b**) histogram of the differences.

**Figure 7:**
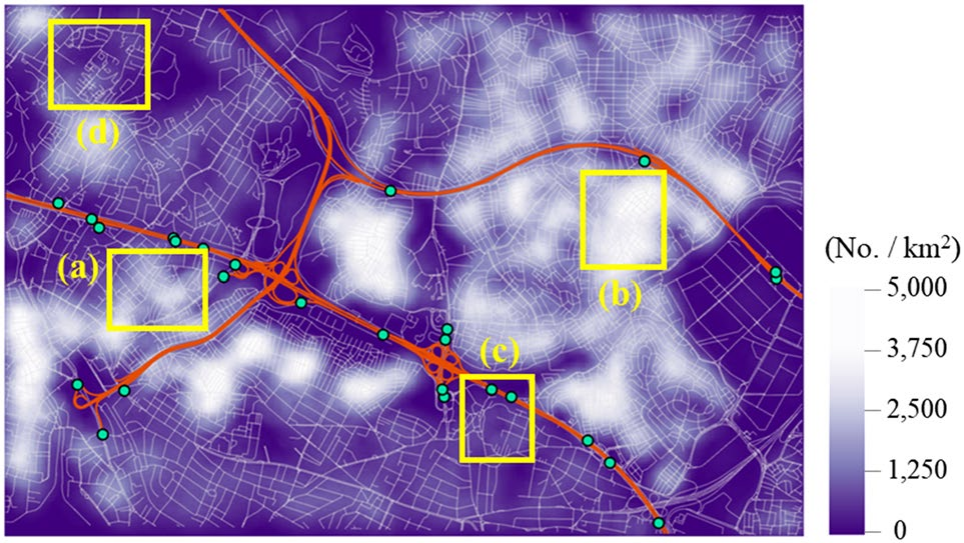
Interpolated density of buildings and location of in depth analysis areas (a) to (d).

To investigate the impact of indirect disruptions, i.e. traffic disruptions caused by debris, a second analysis is performed considering only the direct disruptions, i.e. degradation caused by the structural damage of roadways. The results are obtained as presented in Fig. 4b, while the shifted distribution of $\widehat{E}\left[{RL}_{i}\right]$ is illustrated in Fig. 5a with a dashed orange line. To quantify the difference between the two maps in Fig. 4, their percentage difference is calculated in Fig. 6a, whose values are distributed as illustrated in Fig. 6b. The evaluated difference suggests that while the average difference remains relatively low at 1.87%, the indirect disruptions can have a significant impact, leading to up to 14.6% difference in the resilience loss measure across selected areas. As it can be seen from Fig. 6a, such differences are clustered and not necessarily a result of distance from a highway entrance. Further analysis reveals that the density of buildings is found to have a meaningful impact on system resilience as their interpolated density, which is illustrated in Fig. 7 (the density has been interpolated by counting the number of buildings within radius of 150 m, using ArcGIS Pro software), shows notable correlations with the increase in resilience loss (Fig. 6a). For example, the comparison of Fig. 4a and b reveals that the high resilience loss of the southwestern region is mainly owing to indirect disruptions. Therefore, the resilience of this region can be effectively enhanced by addressing the high density of buildings rather than retrofitting roadway structures. It should also be considered that a high rate of building collapses (indirect disruption) also implies higher casualties rates and hence greater need for access by the emergency services in the immediate aftermath of the event, access which can be severely impaired by the debris obstructing the roads.

On the other hand, building types are found to have low impact on system resilience. To illustrate this, four example locations are selected as marked with yellow boxes in Figs. 4, 6a and 7, and the number of each building type in these areas is summarised in Table 1. While areas (a) and (d) have high proportions of reinforced concrete and steel buildings, which are assigned the most robust fragility curves, such high proportions do not necessarily lead to lower impact of indirect disruptions (Figs. 4b and 6a). It is noted that in Table 1, a larger count does not imply a higher building density since the example sites have different areas.

**Table 1:** Counts and ratios of building types in the four chosen areas.

% Ratio (counts)	Wooden	Masonry	Reinforced concrete and steel	Tunnel-form	Prefabricated	Total
Area (a)	0.3 (2)	8.4 (58)	91.3 (629)	0.0 (0)	0.0 (0)	100.0 (689)
Area (b)	0.1 (1)	30.1 (407)	68.7 (927)	1.1 (15)	0.0 (0)	100.0 (1350)
Area (c)	0.0 (0)	11.43 (20)	72.6 (127)	16.0 (28)	0.0 (0)	100.0 (175)
Area (d)	0.6 (2)	5.26 (18)	94.2 (322)	0.0 (0)	0.0 (0)	100.0 (342)

**Figure 8:**
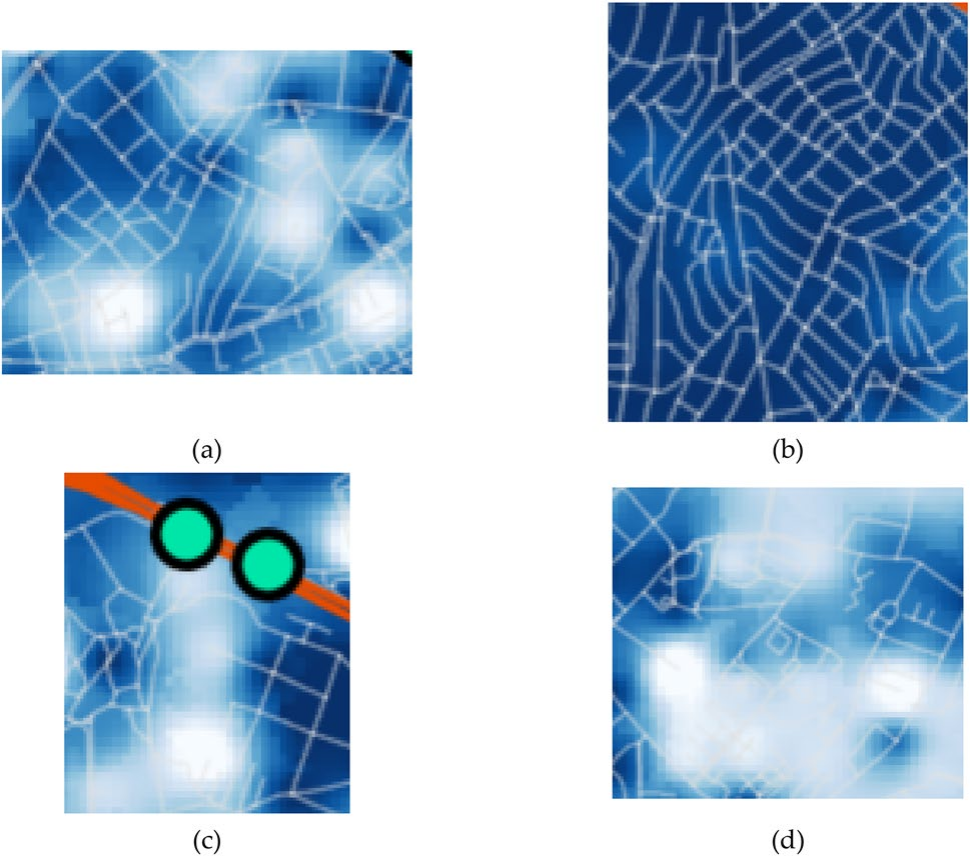
Four example locations in case study area: Maximum flows given undamaged roadways, excerpted from Fig. 3.

**Figure 9:**
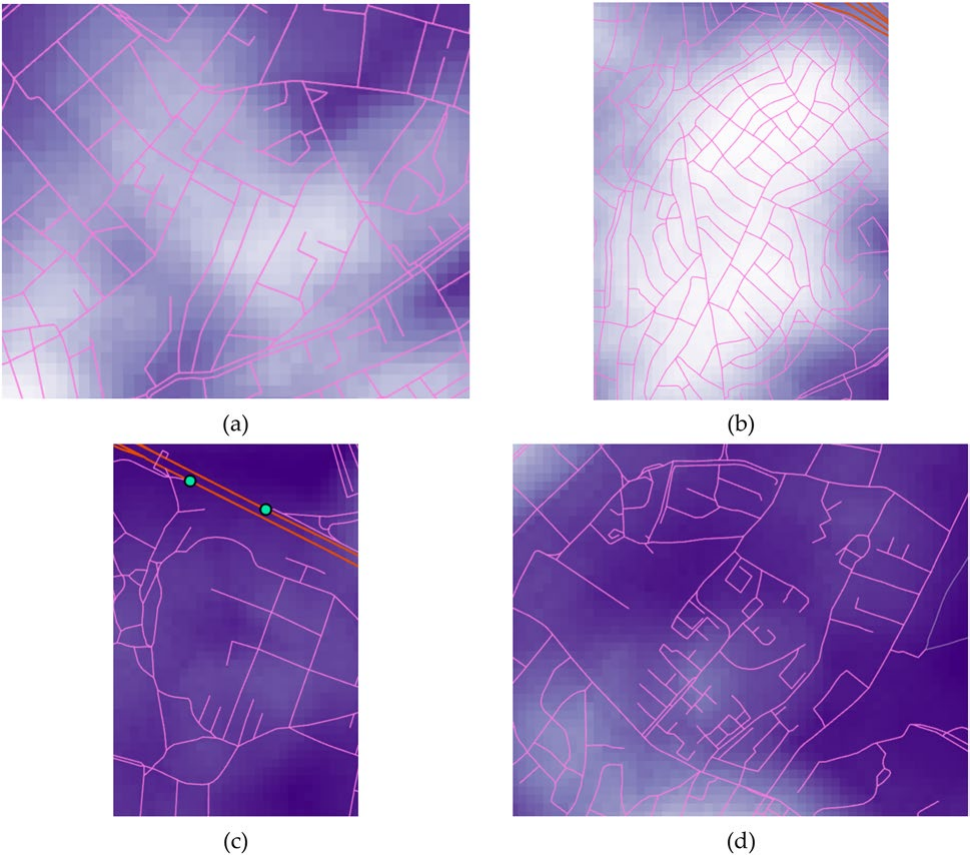
Example locations in case study area: Building density excerpted from Fig. 7.

The investigation suggests that the system resilience is affected mostly by (1) network topology and (2) building density. To understand how different factors influence the system performance, the four example locations are further compared using their enlarged images of analysis results, for maximum flows to destination vertices, whose darker colour implies a better topological efficiency, in Fig. 8, and building densities, whose lighter colour implies higher density, in Fig. 9, respectively. Thus, area (a) has a poorly-connected network topology (with many fragmented roadways) and a high building density, whereby it experiences a high increase in resilience loss (Fig. 6a). On the other hand, area (b) suggests that well-connected roadways (with most roadways having both ends connected to other roads), can overcome high building density and a high proportion of vulnerable buildings (masonry structures up to 30%) as the system resilience loss for the area does not show a notable increase. Meanwhile, a poorly-connected topology can lead to an arbitrary consequence. For example, area (c), which shows a poorly-connected topology and a low density of buildings, experiences a high increase in the resilience loss. In contrast, area (d), which has a similar setting with area (c), does not experience a notable degradation of system performance. However, it shows a high level of overall resilience loss as observed in Fig. 4a, whose main cause seems to be the long distance to destination vertices. Such difference between areas (c) and (d) demonstrates that the consequences cannot be predicted by the separate investigation of individual variables (e.g. network topology, building density and typology, and structural damage of roadways), while underlining the importance of performing system-level analysis that can account for the interplay between individual factors.

## Discussion

This study develops an in-depth analysis of the seismic resilience of a transportation network and applies it to a network located in a sector of the districts Kadıköy and Üsküdar in Istanbul, Turkey. The developed analysis framework innovates on preceding studies in that it accounts for the secondary disruptions of roadways caused by debris from adjacent objects and obtains the analysis result as a map of resilience loss to investigate the presence of disaster inequality across the study area. Thereby, in addition to the distribution of the resilience measure, the analysis results provide comprehensive understandings of the seismic performance of the network including the recovery process, the determinants of system performance and the interplay between analysis variables, which can support decision-making on planning and operating transport infrastructures. It is highlighted that topology of the local road network at neighbourhood level and building density, both play a critical role in the loss of functionality and recovery time for such network. Moreover, the proposed analysis can highlight the dynamic relationship between local network topology and access points to the main arterial network, which is also a primary determinant of the whole network resilience. This has clear implications for urban regeneration plans such as determining new building typologies, footprints and maximum height.

To perform such analysis, a probabilistic model using Bayesian network (BN) is developed to represent the seismic resilience of transportation networks. Then, the BN model is quantified following the proposed data schema. Thereby, a complex analysis can be performed over a large number of variables including seismic hazards, structural damage, recovery process of both structural repair and debris clearance, the functionality of roadways and the system, leading to the determination of a system-level resilience-loss measure. The application to a transportation network in Istanbul demonstrates how various models, assumptions and data can be employed for a large-scale resilience analysis. The proposed BN system and data schema are versatile and therefore can be adopted to reflect the characteristics of a case study network and the purpose of the specific investigation, in this case the accessibility from local sites to arterial roads, evaluated by performing maximum flow analysis.

While the present study provides useful insights, the resilience analysis of real-world systems involves a multitude of models and assumptions, which underlines the need for further research to improve their accuracy, e.g. structural response, network analysis including traffic demand and local inventory data. In particular, more efforts are required to better understand the traffic disruptions by adjacent objects collapsing onto roadways for which only a few references and data are available. Such research topics also include varying debris impacts depending on structural types and debris clearance/repair process. On the other hand, since it is inevitable to introduce a set of assumptions to address the knowledge gap, sensitivity analysis and uncertainty quantification should be employed to grasp and control their impact on analysis results. Similarly, model validation of community-level resilience analysis through real data is also a crucial and open question. Finally, defining and estimating the disaster resilience of transportation systems deserves a thorough investigation, which should be representative of their multifaceted roles, e.g. the connectivity within a community or the accessibility to critical destinations such as shelters, hospitals and major roads.

## Methods

### Seismic risks in transportation networks

The scope and domain of the seismic resilience analysis of transportation networks is summarised in Table 2, together with the relevant assumptions adopted in this study. Specifically, here, the system refers to a transportation network, which is modelled as an abstract graph that includes the roadway structures as edges and their intersections as vertices. The system functionality is evaluated following the chosen definition of performance measure and the corresponding network analysis tool. As the system performance depends on the functionality of the roadways, the system analysis considers only the edges to be components (i.e. failure of vertices is not considered). On the other hand, the disruption of roadways would be caused by the damage inflicted on the associated structures, which include paved roads, bridges and roadside objects, e.g. buildings and slopes. Such disruption can occur in two ways: (1) the damage of roadway structures, i.e. bridges (as roadways) and paved roads, and (2) debris generated from adjacent structures, i.e. bridges (as overpasses) and roadside objects. Finally, in order to evaluate the seismic damage of these structures, seismic hazards are assessed by two events, ground motion (GM) and the subsequent permanent ground displacement (PGD). While the hazard intensity measures related to GM (e.g. peak ground acceleration (PGA) and spectral acceleration (Sa)) are often used to evaluate the damage of bridges, buildings and slopes, PGD is necessary to assess the failure of paved roadways, which remain insensitive to GM.

**Table 2:** Proposed analysis scope.

Category	Scope	Principal assumption
System	Transportation network	Edges and vertices respectively correspond to roadways and their intersections
Components	Edges	The failure of vertices is not considered
Structures	Paved roads Bridges Adjacent objects	Traffic flow can be disrupted by damaged roadway structures and/or debris from adjacent structures
Seismic hazards	Ground motion Ground displacement	Ground displacement is considered as the secondary hazard of ground motions

### Bayesian network (BN) for seismic resilience analysis of transportation networks

BN is a probabilistic graphical model that represents a probability distribution using a directed acyclic graph (DAG), i.e. the graph has no directed cycles^23^. BN represents each random variable (r.v.) by a node and uses directed arrows to denote directional dependence between the r.v.’s. Although directional dependence does not necessarily imply a causal relationship, a causal relationship is an instance of directional dependence. Such association with causality makes BN useful for modelling real-world systems as their variables in general show apparent causal relationships^13,14^. By supporting the use of such qualitative knowledge (i.e. real-world causal relationship) based on the sound probability theory, BN enables us to set up a complex probability distribution even when available data are insufficient, which is mostly the case for extreme hazard events.

Another advantage of BN is that it breaks down the quantification of a high-dimensional joint distribution into that of individual nodes, which represent much lower-dimensional distributions. For system resilience analysis, such high dimensionality would arise from either the variety of variable types that affect system functionality, the large number of components that constitute a system, or multiple instances representing the time span from a hazard occurrence to a full recovery. By using BN, such a high-dimensional distribution can be factorised into lower-dimensional ones (whose distributions are much easier to quantify) that would then represent local models or data of the interaction between a few variable types, the functionality of a single component, or the status at a specific time window. This also enables us to circumvent the issue of model validation by providing a solid theoretical ground to assimilate local models and data.

**Figure 10:**
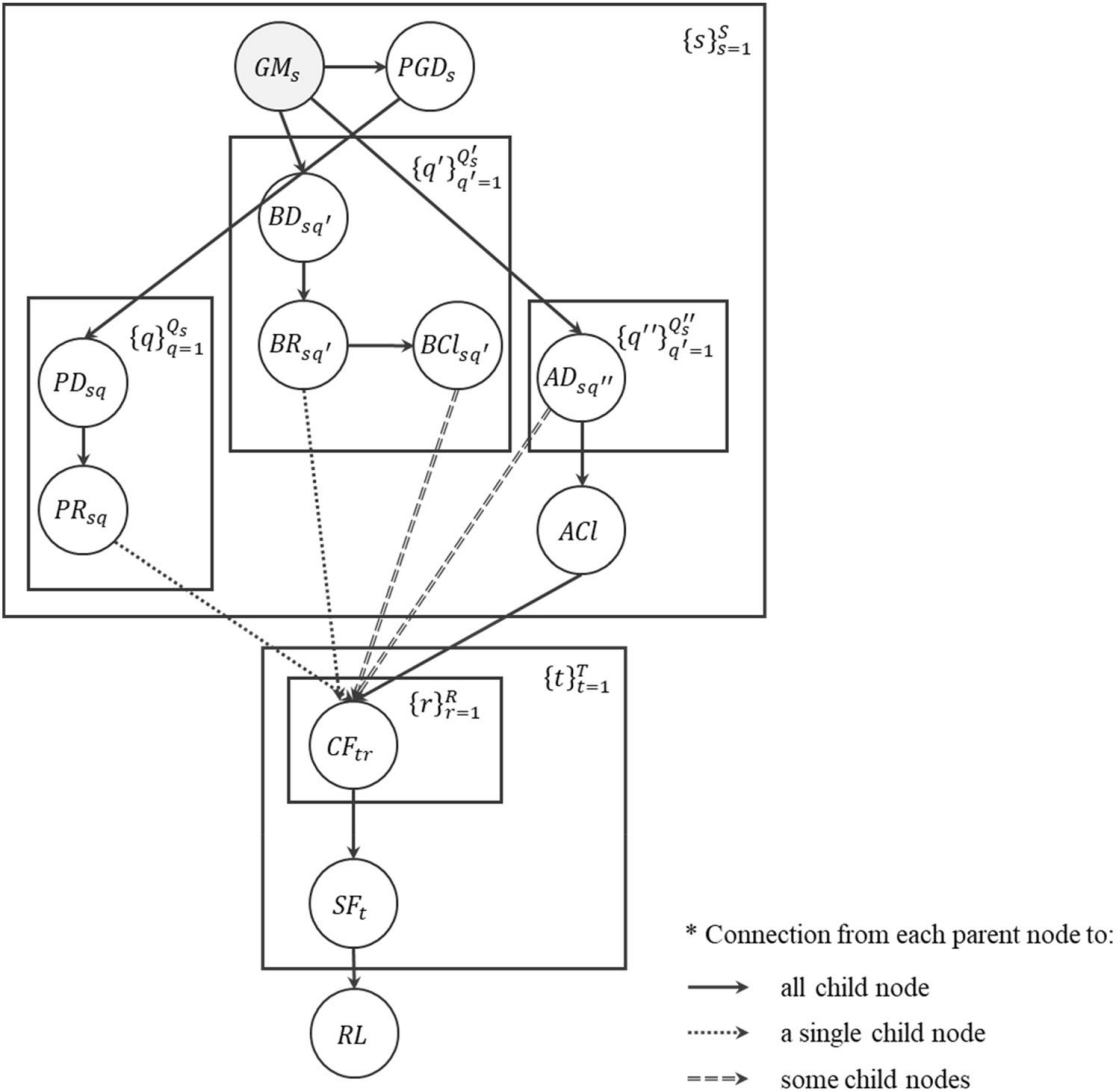
The proposed BN for the seismic resilience analysis of transportation networks.

A BN graph corresponding to the identified scope in Table 2 is established as in Fig. 10. In detail, the hazard events at site *s,* $s=1,\ldots ,S,$ are represented by the nodes $G{M}_{s}$ and $PG{D}_{s},$ where *S* is the number of sites of interest. The two node types represent the IMs that are respectively related to ground motions and ground failures. The node $G{M}_{s}$ is shaded to denote that the IMs of ground motions are considered being deterministic, for which local deterministic hazard maps can be employed. Such assumption of deterministic ground motions is based on two rationales. First, the analysis aims to support the local authorities’ decisions on urban planning and infrastructure investment and therefore, should be aligned with their design hazard map or scenario^24^. Second, and more important, the uncertainty in hazard occurrence and spatial distribution is generally much greater than the uncertainty in robustness and recovery of the system, whereby considering hazard uncertainty is bound to obscure the uncertainties in structural damage and recovery processes, which are of genuine interest for mitigation and resilience^25^. In other words, to perform the probabilistic analysis over the performance of structures, it is proposed to disregard the uncertainty in $G{M}_{s}.$ It is noteworthy, though, that if the probabilistic treatment of ground motions is needed, the analysis can be repeated for different hazard intensity measures with a given probability of occurrence.

Then, the physical damage that is experienced by the structures at site *s,* $s=1,\ldots ,S,$ is represented by the nodes $P{D}_{sq},$ $q=1,\ldots ,{Q}_{s};$ $B{D}_{s{q}^{{\prime}}},$ ${q}^{{\prime}}=1,\ldots ,{Q}_{s}^{{\prime}};$ and $A{D}_{s{q}^{{{\prime\prime}}}},$ ${q}^{{{\prime\prime}}}=1,\ldots ,{Q}_{s}^{{{\prime\prime}}}.$ Each set accounts for the initial damage state of paved roads, bridges and adjacent objects, respectively, while the constants ${Q}_{s},$ ${Q}_{s}^{{\prime}}$ and ${Q}_{s}^{{{\prime\prime}}}$ denote the number of paved roads, bridges and adjacent objects within the site *s*. On the other hand, different groups of structures are associated with different processes of recovery. At site *s*, $P{R}_{sq}$ represents the structural repair process of paved road *q*; and for bridge *q’,* $B{R}_{s{q}^{{\prime}}}$ and $BC{l}_{s{q}^{{\prime}}}$ stand for the repair works and debris clearance, respectively. In contrast, the BN model assumes that the clearance of debris generated from adjacent objects is undertaken globally, which is taken into account by node $ACl$ that is not associated with index *s*.

At time $t,$ $t=1,\ldots ,T,$ the functionality of an edge *r* and of the system are represented by the r.v.’s $C{F}_{tr},$ $r=1,\ldots ,R,$ and $S{F}_{t},$ respectively, where $T$ and $R$ denote the total analysis time in days and the total number of edges. Finally, the node $RL$ stands for the system’s resilience loss.

In the graph, the solid arrows indicate the connection to all child nodes. For example, the hazard node $G{M}_{s}$ is connected to nodes $B{D}_{s{q}^{{\prime}}}$ and $A{D}_{s{q}^{{{\prime\prime}}}}$ of all ${q}^{{\prime}}$ and ${q}^{{{\prime\prime}}},$ as all bridges and adjacent objects subjected to collapse are affected by the ground motions at the corresponding site; and $PG{D}_{s}$ is connected to $P{D}_{sq}$ of all $q,$ as the damage of paved roads depends on ground failure at each site. Similarly, all nodes $C{F}_{tr},$ $r=1,\ldots ,R,$ are connected to $S{F}_{t}$ as the system performance depends on all edges; and all $S{F}_{t},$ $t=1,\ldots ,T,$ are connected to $RL,$ which is measured from the system performance integrated over the analysis time. Likewise, node $ACl$ is connected to all $C{F}_{tr}$ under the assumption that the clearance work takes place globally and thereby, affect all roadways.

On the other hand, the dashed (double-dashed) arrows heading to the component node $C{F}_{tr}$ indicate the correspondence to a single $r\in \left\{1,\ldots ,R\right\}.$ Specifically, the nodes related to the disruptions by debris, i.e. $BC{l}_{s{q}^{{\prime}}}$ and $A{D}_{s{q}^{{{\prime\prime}}}},$ can be connected to $C{F}_{tr}$ of multiple *r*’s for debris from a single structure can affect multiple edges. It is noted that in contrast to $P{D}_{sq}$ and $B{D}_{s{q}^{{\prime}}},$ nodes $A{D}_{s{q}^{{{\prime\prime}}}}$ need to be connected to $C{F}_{tr}$ because of the assumption on the global process of $ACl,$ i.e. the arrows from $A{D}_{s{q}^{{{\prime\prime}}}}$ provide the local damage information. Meanwhile, each of the nodes that represent the time to recover from direct structural damage, i.e. $P{R}_{sq}$ and $B{R}_{s{q}^{{\prime}}},$ always has effect on a single edge; and accordingly, each has exactly one of $C{F}_{tr},$ $t=1,\ldots ,T,$ as their child node.

In order to perform the seismic resilience analysis of transportation networks, the proposed BN can be used as a template of data collection, while, as discussed above, local modifications can be made to reflect variations in analysis scope. Specifically, the BN can be quantified using the hazard map of ground motions ($G{M}_{s}$) associated with return period and probability of occurrence, the model of ground displacements given a ground motion ($PG{D}_{s}$), fragility curves ($P{D}_{sq},$ $B{D}_{s{q}^{{\prime}}},$ and $A{D}_{s{q}^{{{\prime\prime}}}}$), recovery models ($P{R}_{sq}$ and $B{R}_{s{q}^{{\prime}}}$), debris clearance models ($BC{l}_{s{q}^{{\prime}}}$ and $ACl$), traffic disruption models of roadways ($C{F}_{tr}$), the functionality measure of transportation networks ($S{F}_{t}$) and the definition of resilience ($RL$).

On the other hand, as the BN involves a large number of r.v.’s following different types of distributions, it is infeasible to perform exact inference, and therefore, it is proposed to conduct the inference by Monte Carlo simulation (MCS). The MCS should be applied to all r.v.’s, which can be readily addressed by performing ancestral sampling (also called forward sampling), i.e. a realisation is generated by sampling sequentially from parent nodes to child nodes^23^. While MCS can be inefficient when the event of interest is very rare and thus, to be observed, requires a large number of samples, this is irrelevant to the proposed analysis whose result is obtained as resilience loss. In other words, instead of trying to monitor the occurrence of extreme events, this study obtains the result as the distribution of resilience loss values, while presuming the occurrence of an earthquake according to a given hazard map (recall that node $G{M}_{s}$ is considered as being observed). Thereby, the required number of realisations can remain at practical levels even for real-world problems, e.g. 1000.

## Data acquisition, data harmonisation and BN quantification

This section illustrates the models and formulas employed to quantify the proposed BN, while details on employed datasets can be found in the accompanied Supplementary Information. For the definition and measures of system functionality and resilience, readers are referred to “Case study network” section.

### Hazard occurrence

The first type of hazard nodes, $G{M}_{s}$ that stand for the ground motions at site *s*, $s=1,\ldots ,S,$ take the values standing for three IMs: peak ground acceleration (PGA), and spectral accelerations (Sa’s) at periods 0.2 s and 1 s. As illustrated in the previous section, these r.v.’s are considered deterministic, and their values have been obtained from a seismic hazard map provided by IMM. The hazard map used in this study has the resolution of 420 m × 550 m, leading to a 15 by 8 grid for the width and length of the case study area, i.e. in the proposed BN, the number of sites becomes $S=15\times 8$. The second type of hazard nodes, $PG{D}_{s},$ which represent the ground failures, take their values as PGD. The distribution $P\left(PG{D}_{s}|G{M}_{s}\right)$ is evaluated following the procedure provided by *HAZUS-MH*^26^. Because of the absence of data to estimate the ground susceptibility of the study area, the evaluation is conducted by using the standard parameters proposed in *HAZUS-MH*^26^. The evaluation procedure is summarised in Appendix A of Supplementary Information.

### Damage and recovery of structures

Given the value of the associated hazard node, the initial damage nodes, i.e. $P{D}_{sq},$ $B{D}_{s{q}^{{\prime}}}$ and $A{D}_{s{q}^{{{\prime\prime}}}},$ can be quantified using the fragility curve of structure *q*. Fragility curves are in general developed as lognormal distributions, identified by a mean ${m}_{D{S}_{k}}$ and standard deviation ${\beta }_{D{S}_{k}}$ for each damage state $D{S}_{k},$ $k=1,\ldots ,K,$ where $K$ is the total number of damage states considered. This leads to the probability of each *q* being in or exceeding $D{S}_{k}$ as

4 $$P\left(D{S}_{k}|IM\right)=\Phi \left(\frac{1}{{\beta }_{D{S}_{k}}}\text{ln}\left(\frac{IM}{{m}_{D{S}_{k}}}\right)\right),$$

$\Phi \left(\cdot \right)$ is the cumulative distribution function (CDF) of the normal distribution; and $IM$ denotes the given IM value of either $G{M}_{s}$ or $PG{D}_{s},$ depending on the given structural elements. Using Eq. (4), distributions $P\left(P{D}_{sq}|PG{D}_{s}\right),$ $P\left(B{D}_{s{q}^{{\prime}}}|G{M}_{s}\right)$ and $P\left(A{D}_{s{q}^{{{\prime\prime}}}}|G{M}_{s}\right)$ can be quantified for each structure at each site *s*. While the adjacent objects can include different types of entities such as buildings and slopes, no slopes subjected to landslides have been identified in the case study area, and therefore, only buildings are regarded as the potential source of debris.

The fragility curves have been selected from the database compiled by SYNER-G project, a European Collaborative Research Project (conducted from 2009 to 2012) on seismic risk of buildings, lifelines and infrastructures^27^. One of their achievements was to collect a comprehensive set of fragility curves that are applicable especially for the European environment, and therefore the dataset is suitable for the case study area which is located in Turkey. The selected curves are defined in terms of different IMs depending on structural types: Paved roads are vulnerable to PGD; the damage of bridges is evaluated by Sa at period 1.0 s; and buildings (i.e. adjacent objects) are assessed by spectral displacement (Sd). Fragility curves are selected such that they can account for all types of structures in the case study area, according to which the IMs are determined (cf. Appendix C in Supplementary Information). The value of Sd, which is not available from the obtained hazard map, is evaluated from Sa’s at periods 0.2 s and 1.0 s. To this end, the procedure presented by the  *SYNER-G* project^27^ is used, as summarised in Appendix B of Supplementary Information.

Each structure is assigned a fragility curve based on its structural properties, for which the inventory data of roadways and buildings have been obtained from IMM. The data provides information on 10,318 roadways, which are, using the satellite imagery from Google Earth, classified as either a paved road or a bridge. Paved roads are then further classified into either urban or major roads based on their speed limit (i.e. those with speed limit less than 50 km/h are classified as urban roads, and otherwise, they are considered major roads). Within the study area, 54 bridges have been identified and classified within several structural types. As clearly outlined in literature their specific seismic fragility depends on interplay of structural components (e.g. columns, piers decks and bearings), and therefore there is a need for component identification and performance analysis^28,29^. Accordingly, a preliminary study^30^ collated all relevant parameters of the bridges in the case study area by investigating design documents and, where such data was unavailable, referring to satellite imagery provided by Google Earth. In this study, fragility curves are assigned to each bridge by following the parameters summarised in Table A.3–9 in Resvanis^30^ (the information can also be found in Table 5.1–5.9 in D'Ayala et al.^31^). Meanwhile, the dataset supplied by IMM includes 36,921 buildings within the study area, whose structural types are classified by construction type (i.e. masonry wall, wooden frame, reinforced concrete tunnel form (wall and slab), reinforced concrete frame and steel frames), construction year and the number of storeys. The fragility curves selected for each structure and typology are summarised in Appendix C of Supplementary Information.

The nodes related to structural repair, i.e. ${PR}_{sq}$ and $B{R}_{s{q}^{{\prime}}},$ take their values as the number of days required to repair the corresponding structures. Their distributions, i.e. $P\left({PR}_{sq}|P{D}_{sq}\right)$ and $P\left(B{R}_{s{q}^{{\prime}}}|B{D}_{s{q}^{{\prime}}}\right),$ have been evaluated using the statistical model provided by *HAZUS-MH*^26^ as illustrated in Appendix D of Supplementary Information. On the other hand, the debris clearance work for bridges is accounted by nodes $BC{l}_{s{q}^{{\prime}}},$ for which it is assumed that the roadways underneath the corresponding bridge are blocked during the first 10% of the recovery days, i.e. the value of $B{R}_{s{q}^{{\prime}}}.$ For the debris clearance of the adjacent objects (i.e. buildings for the case study), the clearance process is represented by the node $ACl,$ for which the debris amount is estimated using the number of floors and the damage state. To this end, among five damage states of buildings (i.e. none, slight, moderate, extensive and complete), only extensive and complete states are assumed to incur debris amounting to half and all of the number of stories, respectively. Then, the number of days required to clear the debris is estimated by assuming a clearance rate of 200 floors/day over the study area, which results in the clearing work taking around 10 days on average (a discussion on uncertainties associated with the problem of debris clearance can be found in Celik et al.^32^). While the clearance rate can take various units, e.g. number of buildings/day and area/day, floors/day has been chosen for consistency with the assumed debris generation model. With all these settings, the distributions $P\left(BC{l}_{s{q}^{{\prime}}}|B{R}_{s{q}^{{\prime}}}\right)$ and $P\left(ACl|{\left\{A{D}_{s{q}^{{{\prime\prime}}}}\right\}}_{\forall s,{q}^{{{\prime\prime}}}}\right)$ can be quantified. It is noted that while the clearance rate can be chosen depending on local conditions, the assumed value governs the recovery trend during the initial period in contrast to the parameters of structural repair which determines long-term recovery processes.

It is noted that while the nodes related to debris clearance is assumed deterministic primarily because of the lack of data and models, the proposed BN can handle their uncertainties as well if relevant information is provided. Another potential adaptation is to design the model with time-dependent variables since the repair work pace is expected to vary as the network recovers.

### Traffic capacity of edges

The traffic capacity of an edge, i.e. its component functionality, given no structural damage nor disruptions by debris, is evaluated by the number of lanes and speed limit, based on the simplified formulations provided by Transportation Research Board^33^. Given the number of lanes $N{L}_{r}$ and speed limit ${S}_{r}$ of an edge $r,$ the edge with ${S}_{r}\le 50 \,\text{km}/\text{h}$ is assumed to have interrupted traffic flows, whose traffic capacity $E{F}_{r}$ is evaluated as

5 $$E{F}_{r}=\text{1800}\cdot \left(N{L}_{r}-1+{p}_{0,r}^{*}\right),$$

where ${p}_{0,r}^{*}$ refers to the probability that there will be no queue in the road, and is assumed, in this study, as ${p}_{0,r}^{*}=0.2+0.6\cdot \left({S}_{r}-5\right)/\left(70-5\right).$ On the other hand, the edges with ${S}_{r}>50 \text{km}/\text{h}$ are regarded as multilane highways, whose traffic capacity per lane is interpolated (or extrapolated if needed) linearly by comparing ${S}_{r}$ with the speed limit and capacity summarised in Table 3 of Transportation Research Board^33^. To obtain the total capacity, the interpolated value is multiplied by $N{L}_{r}.$

**Table 3:** Capacity of multilane highways depending on speed limit^33^.

Speed limit (mi/h)	Speed limit (km/h)	Capacity (veh/h/lane)
70	113	2300
65	105	2300
60	96.6	2200
55	88.5	2100
50	80.5	2000
45	72.4	1900

Compared to models employed for BN quantification of other r.v.’s in this study, very few models and data are available to quantify the degradation and recovery of an edge’s traffic capacity either caused by structural damage^34^ or by disruptions owing to debris generation^6,35^. To overcome such lack of relevant information, in the following illustrations, we adopt one of the feasible and most common approaches: the level of functionality is divided into a few discrete states by referring to damage states. Specifically, the reduction in an edge’s capacity caused by the damage of the corresponding roadway structure, is evaluated as 0% and 50% of the undamaged capacity, respectively during the first and the second half of the period required for structural repair; after that, they are assumed as recovered to their full capacity. In regard to the disruption caused by the debris from overpass bridges, the underpass edge is assumed to be completely blocked during the days taken to clear the debris.

For debris generated from buildings, it is assumed that any building with its centroid at a distance less than 16 m from the roadway can cause obstructions. The distance has been chosen by observing that in the case study area many buildings directly face the road, leaving minimum clearance between their façades and roadways. In alignment with Anelli et al.^35^, each building is assumed to generate debris either in all of the four directions or in one direction, each with probability 0.5; if it falls in one direction, one of the four directions is selected with a uniform probability. The building will block the roadway in the selected direction, by one lane (all lanes) in case of extensive (complete) damage state. Then, assuming that the debris clearance takes place simultaneously over the affected area, the number of closed lanes of an edge is assumed to decrease proportionately to the daily rate of debris clearance (i.e. the inverse of the days taken for debris clearance).

To determine the value of $C{F}_{tr},$ among the multiple capacity values evaluated from different disruption mechanisms, i.e. the roadway’s damage and fallen debris from associated structures, the smallest value is chosen as the final capacity. As illustrated so far, the current study assumes a deterministic relationship between the damage states of structures and the traffic capacity of edges since there are not enough data to model the uncertainty. However, especially given relevant reconnaissance data and simulation models, one may prefer to define the relationship probabilistically.

## Supplementary Information

Supplementary Information.

## Data Availability

The datasets generated during and/or analysed during the current study are available from the corresponding author on reasonable request.
