# Supplementary material for: Urban seismic resilience mapping: a transportation network in Istanbul, Turkey
Source: Sci Rep. 2022 May 17;12:8188. doi: 10.1038/s41598-022-11991-2 (PMC9114029; doi:10.1038/s41598-022-11991-2)
Supplement: Supplementary file 1 — Supplementary Information. [file 41598_2022_11991_MOESM1_ESM.docx]

Appendix – Urban seismic resilience mapping: a transportation network in Istanbul, Turkey

Ji-Eun Byun ^1^* and Dina D’Ayala ^2^

^1^ Engineering Risk Analysis Group, Technical University of Munich, Munich, Germany; j.byun@tum.de

^2^ Department of Civil, Environmental and Geomatic Engineering, University College London, London, United Kingdom; d.dayala@ucl.ac.uk

***** Correspondence: j.byun@tum.de; Tel.: +49-89-289-23049

Appendix A. Evaluation of permanent ground displacement by landslides

Given an intensity measure of ground motion at site *s*, i.e. $GM_{s},$ the permanent ground displacement (PGD) by landslides at the site, $PGD_{s}$ is evaluated following the procedures provided in Chapter 4.2.2.2 of [26], i.e.

| $PGD_{s}=\left( d/a_{is} \right)\cdot a_{is}\cdot n$ |  |
| --- | --- |

where $\left( d/a_{is} \right),$ $a_{is}$ and *n* respectively represent the displacement factor (cm/g/cycle), induced acceleration in $g$ and the number of cycles. The bounds on $\left( d/a_{is} \right)$ are provided by [26] which is presented in Figure A-1; and to account for the uncertainty, the uniform distribution is assumed between the upper and lower bounds in the logarithm scale. In the *x*-axis of the figure, $a_{c}$ indicates the critical accelerations (i.e. a landslide is expected to occur if $a_{is}\geq a_{c}),$ whose values for each geologic susceptibility categories are listed in Table A-1. The table also illustrates the reference percentage of the categories suggested by [26], which was used for the current analysis^[[1]](#footnote-2)^ as the geologic information of the case study area is absent. On the other hand, $a_{is}$ is assumed to be equal to the peak ground acceleration (PGA) of the site, while $n$ is evaluated as [26]

| $n=0.3419M_{w}^{3}-5.5214M_{w}^{2}+33.6154M_{w}-70.7692$ |  |
| --- | --- |

for which the moment magnitude $M_{w}$ is assumed to be 7.5.


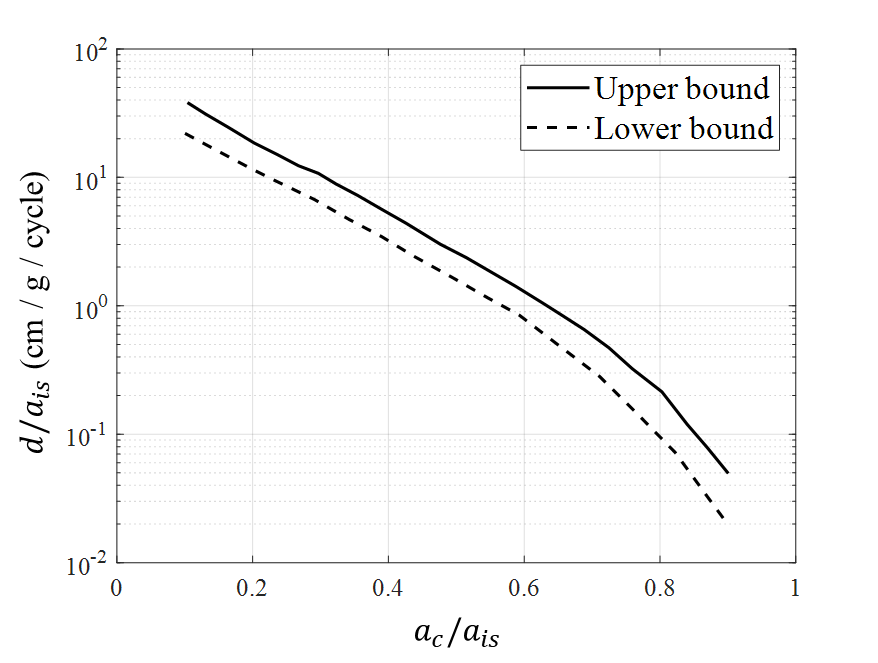


Figure A-1 Bounds on displacement factor $d/a_{is},$ burrowed from *HAZUS-MH* [26]

Table A-1. Critical acceleration ($a_{c}$) and reference percentage of map area for susceptibility categories, burrowed from [26]

| **Susceptibility**  **category** | **None** | **I** | **II** | **III** | **IV** | **V** | **VI** | **VII** | **VIII** | **IX** | **X** |
| --- | --- | --- | --- | --- | --- | --- | --- | --- | --- | --- | --- |
| **Critical**  **accelerations (g)** | None | 0.60 | 0.50 | 0.40 | 0.35 | 0.30 | 0.25 | 0.20 | 0.15 | 0.10 | 0.05 |
| **Map area** | 0.00 | 0.01 | 0.02 | 0.03 | 0.05 | 0.08 | 0.10 | 0.15 | 0.20 | 0.25 | 0.30 |

Appendix B. Evaluation of spectral displacement

As summarised in [27], the spectral displacement (Sd) at site *s* for a period *T*, $Sd_{s}\left( T \right),$ can be evaluated by using the IBC 2006 standardised spectral shape [S1]. First, the spectral acceleration (Sa) at site *s* for period *T* (seconds), $Sa_{s}\left( T \right)$ is evaluated from $Sa_{s}\left( 0.3 \right)$ and $Sa_{s}\left( 1.0 \right)$ as

| $Sa_{s}\left( T \right)=\left\{ \begin{matrix} Sa_{s}\left( 0.3 \right)\cdot\left( 0.4+0.6\cdot T/T_{A,s} \right) & , 0<T<T_{A,s} \\ Sa_{s}\left( 0.3 \right) & , T_{A,s}<T<T_{AV,s} \\ Sa_{s}\left( 1.0 \right) & , T_{AV,s}<T<T_{VD} \\ Sa_{s}\left( 1.0 \right)\cdot T_{VD}/T^{2} & , T>T_{VD} \end{matrix} \right.$ |  |
| --- | --- |

where

| $T_{A,s}=0.2T_{AV,s}$  $T_{AV,s}=Sa_{s}\left( 1 \right)/Sa_{s}\left( 0.3 \right)$  $T_{VD}={10}^{\left[ \left( M_{w}-5 \right)/2 \right]}$ |  |
| --- | --- |

In alignment with Appendix A, $M_{w}$ is assumed to be 7.5. Meanwhile, since the obtained seismic hazard map provides $Sa_{s}\left( 0.2 \right)$ instead of $Sa_{s}\left( 0.3 \right),$ it is assumed that $Sa_{s}\left( 0.3 \right)=Sa_{s}\left( 0.2 \right).$ Such assumption does not undermine the analysis accuracy as the two values in general do not have a significant difference and are identical for $T_{A,s}\leq0.2.$ Then, $Sa_{s}\left( T \right)$ in Eq. (B-1) can be used to evaluate $Sd_{s}\left( T \right)$ by the conversion equation

| $Sd_{s}\left( T \right)=Sa_{s}\left( T \right)\cdot\left( T/2\pi\right)^{2}$ |  |
| --- | --- |

Appendix C. Fragility curves of bridges, paved roads and buildings

SYNER-G project [27] collected a comprehensive database of fragility curves over various structural types including bridges, paved roads and buildings, while especially focusing on the environments of Europe. Such Europe-based compilation makes it suitable for the case study as the case study area, i.e. Istanbul, is located in Turkey. Among the compiled data by [27], this study has chosen the ones developed by *HAZUS-MH* [26], whereby the definitions of damage states and intensity measures can remain consistent across different types of structures. The chosen fragility curves are summarised in Table C-1, Table C-2 and Table C-3 for bridges, paved roads and buildings, respectively.

Bridges in the case study area are classified into four classes that are listed in Table C-1, for which the structural parameters are identified following [30]. In the table, the damage states 1, 2, ⋯, 5 respectively stand for the states of none, slight, moderate, extensive and complete damage. The fragility curves of bridges are defined in regard to Sa at 1.0 second, and all curves have the standard deviation as 0.6, i.e. in Eq. (4), $\beta_{DS_{k}}=0.6$ for $k=1,\cdots,5.$ On the other hand, the fragility curves of paved roads are summarised in Table C-2, where a road has four possible damage states, i.e. none, slight, moderate and extensive/complete. The damage states are defined in terms of PGD in inch, while all fragility curves have standard deviation as 0.7, i.e. $\beta_{DS_{k}}=0.7$ for $k=1,\cdots,4.$ Finally, buildings are classified based on construction year, structural type and number of stories, as illustrated in Table C-3. While the buildings are evaluated in terms of Sd, only two damage states are considered during evaluation as illustrated in **Methods** section, i.e. damage states 4 and 5 that respectively stand for extensive and complete damage.

Table C-1. Fragility curves of bridges: structural types and mean values in Sa at period 1.0 second ($g$)

| **Description of structural type** | $\boldsymbol{m}_{\boldsymbol{D}\boldsymbol{S}_{\boldsymbol{k}}}\boldsymbol{,}$ $\boldsymbol{k}\boldsymbol{=}\boldsymbol{1}\boldsymbol{,}\boldsymbol{2}\boldsymbol{,\cdots,}\boldsymbol{5}\boldsymbol{,}$ **in Eq. (4)** |
| --- | --- |
| Single span | [0.00, 0.80, 1.00, 1.20, 1.70] |
| Multi-column bent;  simple support – prestressed concrete | [0.00, 0.50, 0.80, 1.10, 1.70] |
| Single column;  box girder – prestressed continuous concrete | [0.00, 0.60, 0.90, 1.10, 1.50] |
| Non-conventional type | [0.00, 0.80, 1.00, 1.20, 1.70] |

Table C-2. Fragility curves of paved roads: structural types and mean values in PGD (inch)

| **Road type** | $\boldsymbol{m}_{\boldsymbol{D}\boldsymbol{S}_{\boldsymbol{k}}}\boldsymbol{,}$ $\boldsymbol{k}\boldsymbol{=}\boldsymbol{1}\boldsymbol{,}\boldsymbol{2}\boldsymbol{,\cdots,}\boldsymbol{4}\boldsymbol{,}$ **in Eq. (4)** |
| --- | --- |
| Urban road | [0, 6, 12, 24] |
| Major road | [0, 12, 24, 60] |

Table C-3. Fragility curves of buildings: Structural types, periods, and mean and standard deviation values in spectral displacement (inch)

| **Structural type** | | | **Period (sec)** | $\left( \boldsymbol{m}_{\boldsymbol{D}\boldsymbol{S}_{\boldsymbol{k}}}\boldsymbol{,}\boldsymbol{\beta}_{\boldsymbol{D}\boldsymbol{S}_{\boldsymbol{k}}} \right)$ | |
| --- | --- | --- | --- | --- | --- |
| **Construction year** | **Construction**  **type** | **Number of stories** |  | $\boldsymbol{k=4}$ | $\boldsymbol{k=5}$ |
| Before 1980 | Wooden | 1-2 | 0.35 | (3.86, 1.02) | (9.45, 0.99) |
|  |  | 3-5 | 0.40 | (6.62, 0.89) | (16.20, 0.99) |
|  | Masonry | 1-2 | 0.35 | (2.03, 1.10) | (4.73, 1.08) |
|  |  | 3-8 | 0.50 | (3.15, 0.87) | (7.35, 0.91) |
|  | Reinforced concrete,  Steel construction | 1-3 | 0.50 | (4.38, 0.78) | (10.80, 0.96) |
|  |  | 4-7 | 1.08 | (7.30, 0.85) | (18.00, 0.98) |
|  |  | 8+ | 2.21 | (11.38, 0.76) | (28.08, 0.92) |
|  | Tunnel-form building | 4-7 | 0.65 | (5.78, 0.90) | (15.75, 0.99) |
|  | Prefabricated | 1-3 | 0.35 | (2.89, 0.98) | (7.88, 0.96) |
|  |  | 4-7 | 0.56 | (4.81, 0.84) | (13.12, 0.99) |
|  |  | 8+ | 1.09 | (6.93, 0.89) | (18.90, 0.98) |
| Between 1980 and 2000 | Wooden | 1-2 | 0.35 | (3.86, 0.89) | (9.45, 1.04) |
|  |  | 3-5 | 0.40 | (6.62, 0.95) | (16.20, 0.92) |
|  | Reinforced concrete,  Steel construction | 1-3 | 0.50 | (5.08, 0.74) | (12.96, 0.88) |
|  |  | 4-7 | 1.08 | (8.46, 0.69) | (21.60, 0.87) |
|  |  | 8+ | 2.21 | (13.21, 0.71) | (33.70, 0.83) |
|  | Prefabricated | 1-3 | 0.35 | (3.37, 1.03) | (9.45, 0.88) |
|  |  | 4-7 | 0.56 | (5.61, 0.75) | (15.75, 0.93) |
|  |  | 8+ | 1.09 | (8.08, 0.77) | (22.68, 0.89) |
| After 2000 | Wooden | 1-2 | 0.35 | (5.04, 0.85) | (12.60, 0.97) |
|  |  | 3-5 | 0.40 | (8.64, 0.90) | (21.60, 0.83) |
|  | Reinforced concrete,  Steel construction | 1-3 | 0.50 | (6.48, 0.69) | (17.28, 0.72) |
|  |  | 4-7 | 1.08 | (10.80, 0.67) | (28.80, 0.74) |
|  |  | 8+ | 2.21 | (16.85, 0.65) | (44.93, 0.67) |
|  | Tunnel-form building | 8+ | 1.32 | (13.48, 0.69) | (39.31, 0.77) |
|  | Prefabricated | 1-3 | 0.35 | (4.32, 0.98) | (12.60, 0.94) |
|  |  | 4-7 | 0.56 | (7.20, 0.70) | (21.00, 0.82) |
|  |  | 8+ | 1.09 | (10.37, 0.68) | (30.24, 0.81) |

Appendix D. Structural repair

The days required to repair bridges and paved roads are evaluated based on the statistics provided by [26] which is presented in Table D-1. The uncertainty in the number of recovery days is accounted by assuming the normal distribution with the mean and standard deviation provided in the table.

Table D-1. Restoration functions for roadway structures

| **Damage state** | **Paved roads** | | **Bridges** | |
| --- | --- | --- | --- | --- |
|  | **Mean (days)** | **Std. (days)** | **Mean (days)** | **Std. (days)** |
| **Slight** | 0.9 | 0.05 | 0.6 | 0.6 |
| **Moderate** | 2.2 | 1.8 | 2.5 | 2.7 |
| **Extensive** | 21 | 16 | 75.0 | 42.0 |
| **Complete** |  |  | 230.0 | 110.0 |

References

1. International Code Council. *International Building Code (IBC-2006)*, United States. (2006).

1. The given ratios are normalized so that their sum would be 1. [↑](#footnote-ref-2)
